# A Rare Cause of Acute Bilateral Hearing Loss: Otosyphilis

**DOI:** 10.7759/cureus.11243

**Published:** 2020-10-29

**Authors:** Karim Amidou Abdul, Luis Silva, Jorge Perez

**Affiliations:** 1 Internal Medicine, Brandon Regional Hospital, Brandon, USA

**Keywords:** otosysphilis, neurosyphilis, hearing loss

## Abstract

Syphilis is a bacterial infection caused by *Treponema pallidum*. It spreads usually via sexual contact. Syphilis generally presents as a multisystem disease, with symptoms resembling and often confused with those of other diseases, thus often called “the great mimicker”. Neurosyphilis is a rare but late course of the disease process when the meninges and central nervous system (CNS) are involved. Otosyphilis is an even rarer, yet important complication of neurosyphilis and a rare cause of sensorineural deafness often misdiagnosed. We present the case of a 46-year-old Caucasian male admitted for acute onset bilateral hearing loss caused by otosyphilis. We include a discussion about cerebrospinal fluid (CSF) protein analysis in individuals diagnosed with neurosyphilis.

## Introduction

Hearing loss or loss of any sense is a very concerning event for any individual, more so especially when this is sudden. The differential diagnosis to consider for sudden hearing loss is very extensive, including but not limited to acoustic neuroma, immune-mediated disorders, Meniere's disease, vascular alterations and vasculitis, membrane rupture, and infectious agents which can be viral or bacterial in origin [1]. With technological advancements today, establishing the diagnosis and cause of acute hearing loss has become relatively easier. When differentials for acute bilateral hearing loss are considered outside of acute cerebrovascular accidents (CVA), infectious agents such as syphilis as in the case of otosyphilis should be considered. According to the Centers for Disease Control and Prevention (CDC) data, the rate of all stages of syphilis in the United States has been on a rise since around the year 2000/2001 after reaching its historic lowest rate [2]. Otosyphilis is a less recognized complication of syphilis that can lead to irreversible sensorineural hearing loss [3].

## Case presentation

**Figure 1:**
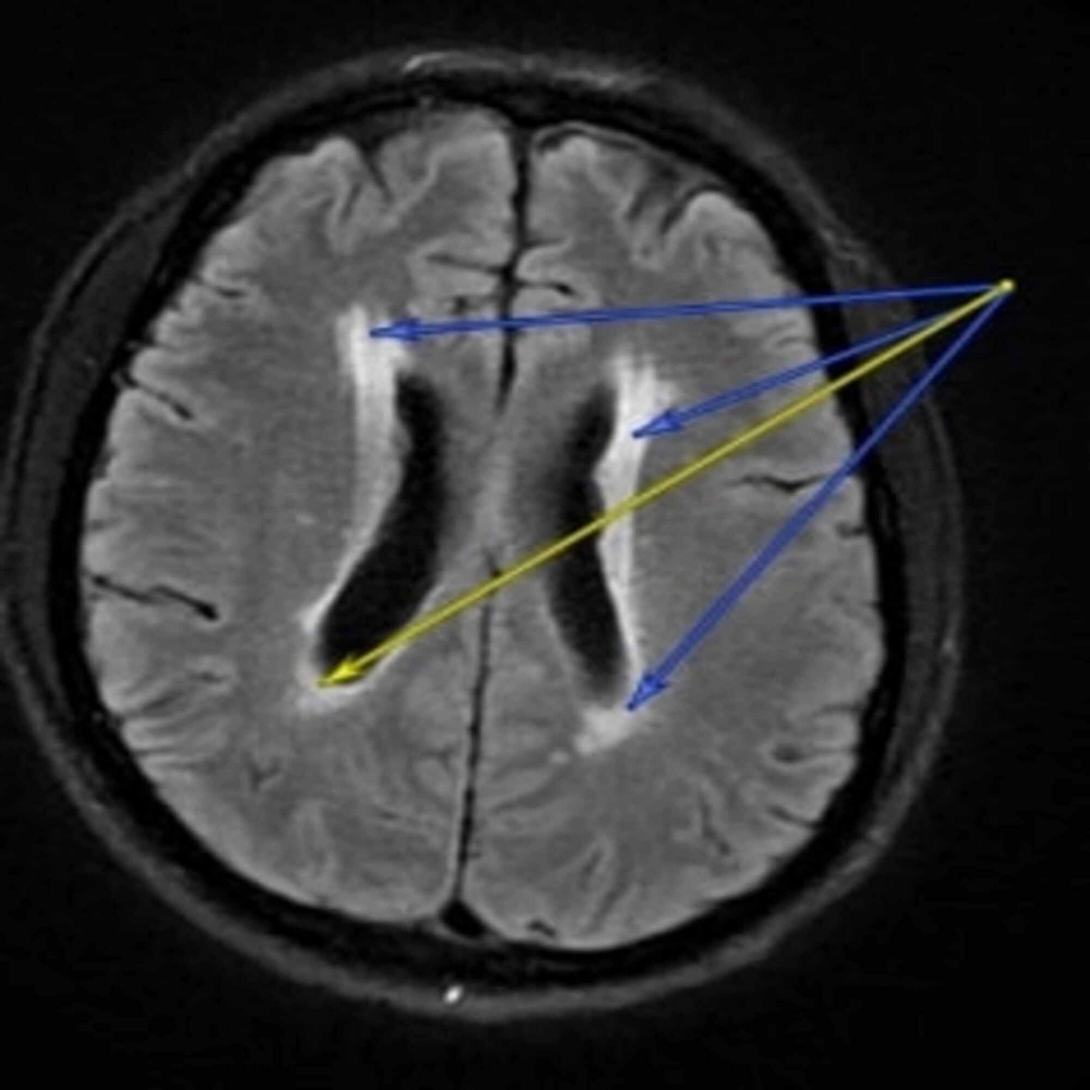
MRI brain with periventricular white matter hyper-intensities. Both yellow and blue arrows showing periventricular hyper-intensity seen on MRI

A 46-year-old male with medical history significant for uncontrolled hypertension admitted to the hospital for acute onset bilateral hearing loss. Symptoms had started four hours prior to presentation while he was watching television. Initial admission computed tomography (CT) scan was negative for any acute infarcts or other intracranial processes. His initial physical exam was significant for mild gait impairment, with Weber and Rinne test consistent with bilateral neurosensory hearing loss. Patient was initially admitted as a possible stroke rule out. Work up for stroke including carotid ultrasound, Cardiac echo were all negative except for Magnetic Resonance Imaging (MRI) which showed some periventricular and deep white matter lesions demonstrating T2 hyper-intensity without enhancement consistent with questionable demyelinating disease as seen in Figure 1. Given the non-specificity of his symptoms and unknown sexual history, a decision was made to evaluate for syphilis. Rapid plasma reagent (RPR) test was ordered, and it came back positive with a 1:16 titer. A confirmatory *Treponema pallidium* fluorescent treponemal antibody absorption-Ab was also positive which confirmed the diagnosis of syphilis. A lumbar puncture was then performed prior to the beginning of treatment and cerebrospinal fluid (CSF) Venereal Disease Research Laboratory (VDRL) was positive with a 1:4 VDRL titer establishing the diagnosis of neurosyphilis. Considering the initial MRI scan in Figure 1 below, was concerning for demyelinating disease, CSF protein analysis and oliclonal band was also obtained. Both CSF protein and oligoclonal band were noticed to be elevated, but in the setting of neurosyphilis and possible meningeal inflammation with increased likelihood for false elevation in CSF protein and negative family history, a decision was made to focus management on neurosyphilis. Patient was started on Penicillin G IV 3mmu (million units) daily for a total period of 14 days, and daily 30mg of prednisone. On day seven of admission after patient had been on penicillin for about three days, he had some hearing improvement. Patient stated it was possible for him to hear himself talk even though he still had some difficulties hearing others. Patients condition remained this way throughout his hospital course and the rest of his treatment was done outpatient.

## Discussion

The definite diagnosis of isolated otosyphilis is challenging and it should be made on the basis of a typical clinical presentation i.e. hearing impairment in the setting of positive serological test results for syphilis. Also, this is a potentially reversible cause of hearing loss if treatment is initiated early. For this reason, overdiagnosis is justifiable. Based on a retrospective review of 85 cases by Kwanchanok et al., comprising 56 males and 29 females, the most common presenting symptoms included hearing loss (90.6%), tinnitus (72.9%), and vertigo (52.9%). The cerebrospinal fluid analysis was positive for VRDL in 5.4% [4]. It is therefore important to understand that positive CSF VDRL and an established diagnosis of neurosyphilis are not required to make a diagnosis of otosyphilis. Even though most cases of otosyphilis are seen in newborns as the congenital form, it has been claimed that up to about 17% of individuals with syphilis can suffer from sensorineural hearing loss [5]. The treatment usually involves extended treatment with penicillin and sometimes adjunct with steroids to help with meningeal inflammation. Usually the response rate to this treatment regimen ranges from 15-80% in congenital neurosyphilis [6]. However, there is conflicting data on the response rate with penicillin in the acquired form of this disease [5]. Some professionals are of the opinion that the management of otosyphilis with penicillin is primarily intended to help arrest progression of the disease as opposed to restoring ability to hear. Overall, it would seem the general consensus is that this disease can be easily treated if caught early otherwise, arrest of progression seems to be the only option [7]. The recommended antibiotic therapy for otosyphilis is the same as for neurosyphilis [6]. Intravenous penicillin for 10-14 days is the recommended treatment. However, ceftriaxone has also shown efficacy when used in multiple-dose regimens, with the most used being 2g IV ceftriaxone for the same duration as penicillin. In addition to intravenous antibiotics, corticosteroids are usually administered in otosyphilis, as this has shown better outcomes and reduced the risk Jarisch-Herxheimer reaction, thus increasing compliance to treatment [6].

## Conclusions

Otosyphilis remains to be a rare cause of hearing loss with varying response to treatment if diagnosed early. It should always be part of the differential diagnosis in patients suffering from sudden sensorineural hearing loss (unilateral or bilateral), fluctuating hearing loss, with tinnitus and vertigo, in which acute cerebrovascular events have been ruled out. In our patient, it is uncertain whether this patient will regain hearing later down the road. Unfortunately tone and speech audiogram was not done for this patient during his hospital stay. It is however recommended to do audiogram during the management of otosyphilis as it helps establishes a baseline and also helps in evaluating the response to the treatment. Moreover, it is important to understand early diagnosis and proper management of this disease prevent progression of the disease and subsequent cardiovascular or ophthalmologic complications which can be really devastating.
